# Organocatalytic Michael Addition of 1,3-Dicarbonyl Indane Compounds to Nitrostyrenes

**DOI:** 10.3390/molecules15042551

**Published:** 2010-04-12

**Authors:** Zhen-Yu Jiang, Hua-Meng Yang, Ya-Dong Ju, Li Li, Meng-Xian Luo, Guo-Qiao Lai, Jian-Xiong Jiang, Li-Wen Xu

**Affiliations:** Key Laboratory of Organosilicon Chemistry and Material Technology of Ministry of Education, Hangzhou Normal University, Hangzhou 310012, China

**Keywords:** organocatalysis, Michael reaction, primary amine, diamine

## Abstract

To map out the efficient organocatalyst requirements in the Michael addition of 1,3-dicarbonyl indane compounds to nitrostyrenes, a dozen different amino organocatalysts containing a *p*-toluenesulfonyl group (Ts) have been evaluated; excellent enantio-selectivities (up to *er* 92:8) were obtained with a primary amine-based Ts-DPEN catalyst and a plausible catalytic reaction mechanism was proposed on the basis of the experimental results.

## 1. Introduction

The addition of stabilized 1,3-dicarbonyl compounds to electro-deficient alkenes is one of the oldest and most useful key bond construction methods in organic synthesis for the formation of new C-C bonds [1]. Catalytic, enantioselective versions of this fundamental transformation which use metal-based chiral complex catalysts or organocatalysts have been reported extensively [2,3,4,5,6,7,8]. Early studies on organocatalytic asymmetric Michael reactions were conducted with readily available amines of natural origin, such as (-)-quinine and (+)-quinidine [9,10,11]. In the case of the Michael reaction of nitrostyrenes, the resulting 1,4-addition product is a very useful precursor to different complex organic molecules, such as amino carbonyl compounds, which is tied to their propensity to undergo facile α-alkylation reaction and interconversions to other important organic functional groups. [12,13] Several attempts have been performed toward achieving asymmetric Michael addition of 1,3-dicarbonyl compounds to nitrostyrenes in the presence of organocatalysts, such as 2-aminobenzimidazole [14], aminothiourea [15,16,17,18,19,20,21,22], guanidine [23,24], primary amine [25], and other amino catalysts [26]. However, the development of the perfect organocatalyst based on the above functionality is difficult because there are many factors that influence enantioselective reactions. Although the enantioselective Michael reaction of ketoesters to nitrostyrenes is a well studied reaction in organocatalysis, the stereocontrolled concurrent creation of adjacent quaternary and tertiary stereocenters still remains challenging. Recently we have reported on the effective enantioselective Michael addition of 1,3-dicarbonyl indane compounds to nitrostyrenes in the presence of Ts-DPEN with primary amine and simple amino *N*-sulfonamide groups [27,28]. To establish whether the primary amine and simple amino *N*-sulfonamide group was important and to map out the structural requirements of organocatalysts, we have now carried out an extensive organocatalyst variation study. For the synthesis of the organocatalysts, our criteria were that the chiral organocatalysts should be derived from similar chiral diamines. Herein, we reported a full account of our investigation focusing on the development of organocatalysts for the Michael addition of 1,3-dicarbonyl indane compounds to nitrostyrenes.

## 2. Results and Discussion

**Scheme 1:**
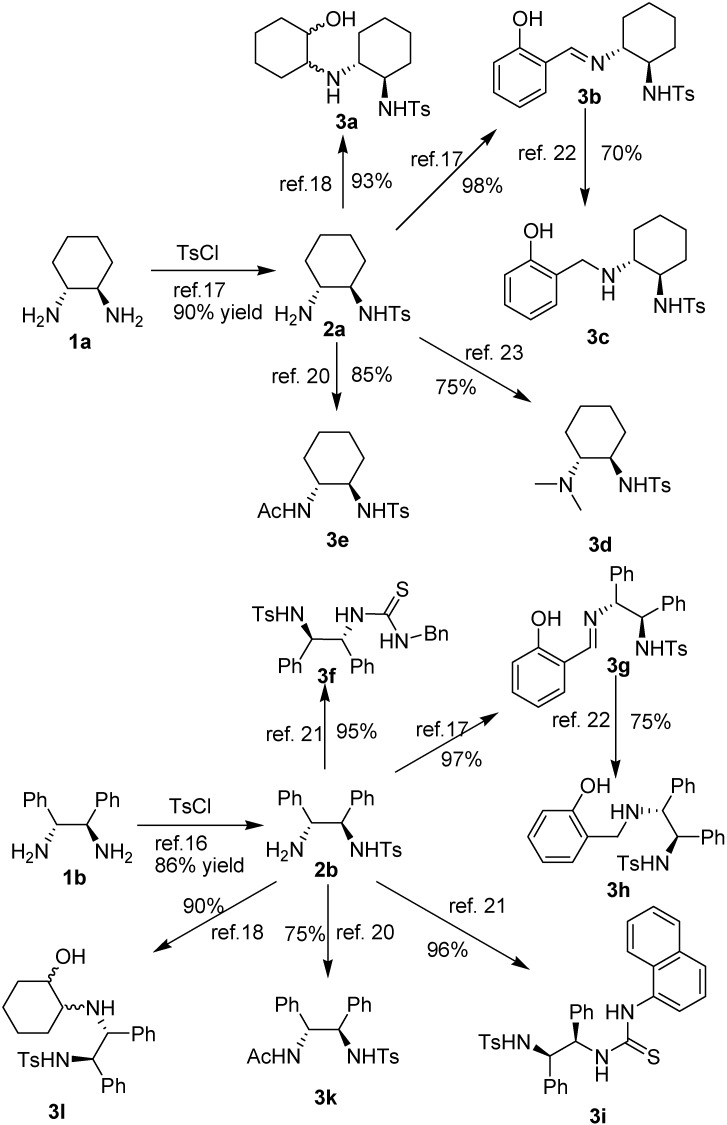
The preparation of different functional organocatalysts derived from chiral diamine.

We designed a series of functional amino analogues with commonly encountered groups, such as thiourea, amide, phenol, tertiary amine and imine, for the catalytic Michael addition of 1,3-dicarbonyl indane compounds to nitrostyrenes. Catalysts **3a-l** containing *p*-methylbenzene sulfonyl groups (Ts) could be easily synthesized in several steps from chiral cyclohexane-1,2-diamine (**1a**) or 1,2-diphenylethane-1,2-diamine (**1b**) (Scheme 1). Chiral diamines **1** reacted with *p*-toluenesulfonyl chloride (TsCl) in DCM at 0 °C to afford the corresponding products **2** in good yield [29,30]. It should be noted that an excess of diamine was necessary to achieve satisfactory yields in this step. Treatment of compounds **2** with Ac_2_O, cyclohexene oxide, salicylaldehyde, and isothiocyanate under reported conditions afforded the corresponding functional catalysts **3** in excellent yields [31,32,33,34]. Catalysts **3c**, **3d**, **3h**, were prepared from **2a** or **2b** by alkylation and reduction [35,36].

**Scheme 2:**
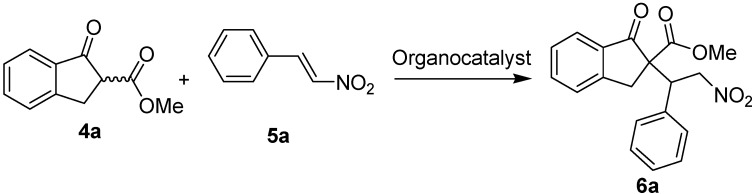
Asymmetric Michael addition of 1,3-dicarbonyl indane compound to nitrostyrene.

**Figure 1:**
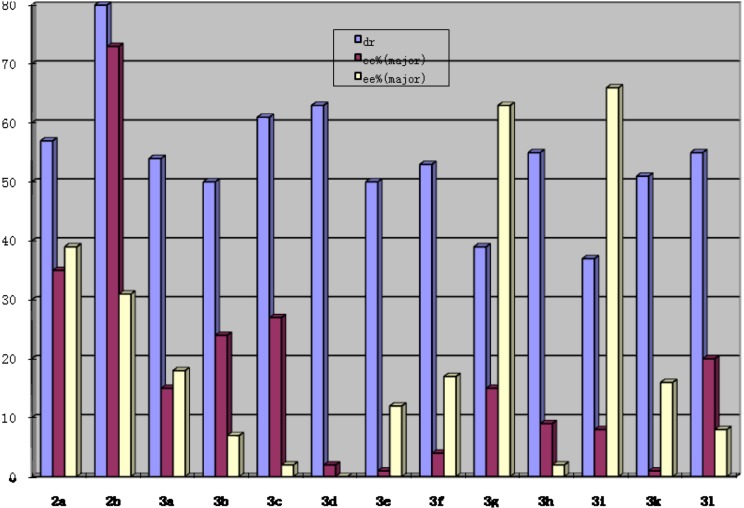
Enantio- and diastereoselectivities in the Michael reaction using different organocatalysts.

To evaluate the different organocatalysts, the following conditions were adopted for the Michael addition of 1,3-dicarbonyl indane compound **4a** to nitrostyrene **5a**: organocatalyst (10 mol%), nitrostyrene **5a** (0.5 mmol) and 1,3-dicarbonyl indane compound **4a** (0.55 mmol) in toluene (1 mL) at -20 °C (Scheme 2). The organocatalysts **3a-l** were evaluated by using the above conditions and full results are shown in Figure 1. The yields are good and similarly for all the cases that obtained after purification by chromatography (70–80%). The *ee* values were determined by using chiral HPLC. In the model Michael addition of 1,3-dicarbonyl indane compound **4a** to nitrostyrene **5a**, the diastereo- and enantioselectivities in the reaction using these organocatalysts varied significantly with different functional substitutes. For example, **3d**, **3e**, or **3h**, has poor enantioselectivity for both enantiomers (*2R* or *2S*), while **3g** or **3i** gave the major *2S*-enantiomer Michael adduct with good enantioselectivity. Interestingly, **2a** containing a similar functional group gave low diastereo- and enantioselectivity in comparison to Ts-DPEN (**2b**) These results suggest that Ts-DPEN (**2b**) is the most suitable Ts-based organocatalyst among these similar amino catalysts in this reaction, and the primary amine group turned out to be a crucial factor to obtain high levels of enantioselectivities.

We next examined the scope of this class of Michael reactions with a series of nitrostyrenes and cyclic *β*-ketoesters under the optimized reaction conditions. As shown in Table 1, in the organocatalytic asymmetric Michael reaction different substituted nitrostyrenes **5** reacted smoothly with methyl 1-oxo-2,3-dihydro-1*H*-indene-2-carboxylate (**4**) with good diastereoselectivities. In this way, it was revealed that major Michael adducts with various nitrostyrenes were obtained in good enantioselectivities (up to 92:8 *er*), while minor *2S*,*2S*-isomers were obtained with moderate enantioselectivities (up to 82:18 *er*). Lower temperature resulted in lower diastereo- and enantioselectivity (Entries 18 and 8), which may be due to the poor interaction between the primary amine group of Ts-DPEN (**2b**) and the 1,3-dicarbonyl compounds at lower temperature. Iinterestingly, methyl 1-oxo-1,2,3,4-tetrahydronaphthalene-2-carboxylate did not react with nitrostyrene under these reaction conditions (Entry 17).

**Table 1:** Enantio- and Diastereoselective Michael Reactions of Nitrostyrenes in the presence of catalytic Ts-DPEN. 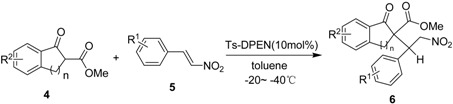

Entry^a^	R^1^	R^2^, n	Time (h)	Yield%^b^	Dr^c^	*er* (*minor*)^d^	*er* (*major*)^d^
1	H	H, n = 1	24	**6a:** 87	80/20	66:34	87:13
2	*p*-Me	H, n = 1	24	**6b:** 85	76/24	60:40	84:16
3	*o*-Cl	H, n = 1	24	**6c:** 81	85/15	64:36	90:10
4	*p*-Br	H, n = 1	24	**6d:** 72	82/18	61:39	87:17
5	*p*-OMe	H, n = 1	24	**6e:** 78	75/25	57:43	86:14
6	*p*-Cl	H, n = 1	24	**6f:** 81	83/17	63:37	86:14
7	2,4-Cl	H, n = 1	24	**6g:** 79	82/18	52:48	85:15
8	*p*-F	H, n = 1	24	**6h:** 85	83/17	65:35	89:11
9	*o*-Br	H, n = 1	24	**6i:** 77	77/23	82:18	92:8
10	*p*-CN	H, n = 1	24	**6k:** 76	85/15	65:35	84:16
11	*o*-Br	4-Me, n = 1	48	**6l:** 72	73/27	71:29	86:14
12	*o*-Br	4-OMe, n = 1	48	**6m:** 87	82/18	75:25	87:13
13	*o*-Br	5-Br, n =1	48	**6n:** 78	75/25	50:50	84:26
14	*o*-Cl	4-Me, n = 1	48	**6o:** 83	76/24	78:22	80:20
15	*o*-Cl	4-OMe, n =1	48	**6p:** 85	84/16	62:38	85:15
16	*o*-Cl	5-Br, n =1	48	**6q:** 87	74/26	50:50	82:18
17	*o*-Cl	H, n =2	48	trace	-	-	-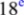	*p*-F	H, n = 1	24	**6h:** 93	57/43	51:49	77/23

^a^ The reactions were performed with 0.5 mmol of nitrostyrene, 0.55 mmol of cyclic *β*-ketoester, 10 mol% of Ts-DPEN, in toluene (1 mL), at -20 °C. ^b^ Isolated yield. ^c^ Determined by ^1^H NMR. ^d^ The er values of major and minor prodcuts were determined by HPLC, and the absolute configuration was not determined. ^e^ At -40 °C.

**Figure 2:**
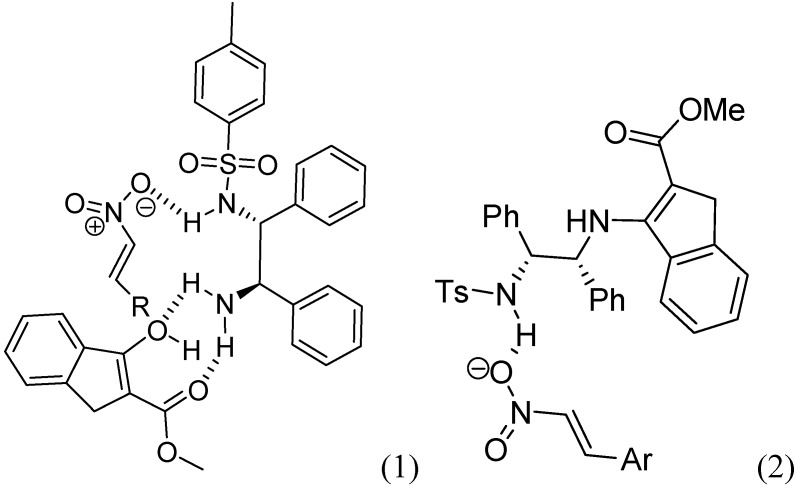
Proposed catalytic reaction mode *via* dual activation model.

On the basis of the experimental results described in this article, a reasonable catalytic reaction model is provided in Figure 2. In the (1), the reaction may proceed by the dual activation model, the carbonyl group of enol intermediate of *β*-ketoester was assumed to interact with primary amine moiety of Ts-DPEN via multiple H-bonds, thus increasing the nucleophilic ability of the reacting carbon center. The H-sulfonamide activates nitrostyrenes via a single hydrogen bond and enhances the electrophilicity of the olefin. However, an alternative mechanism with enamine catalysis is also reasonable [37,38]. As shown in Equation (2), the reaction is preceded with the combination of enamine activation of primary amine and hydrogen-bonding activation of Ts- group.

## 3. Experimental Section

### 3.1. General

All reagents and solvents were used directly without purification. Flash column chromatography was performed over silica (200–300 mesh). ^1^H-NMR and ^13^C-NMR spectra were recorded at 400 and 100 MHz, respectively on Advance (Brucker) 400 MHz Nuclear Magnetic Resonance Spectromer, and were referenced to the internal solvent signals. IR spectra were recorded using a FTIR apparatus on Nicolet 700 spectrophotometer in KBr cells. Thin layer chromatography was performed using silica gel; F_254_ TLC plates and visualized with ultraviolet light. HPLC was carried out with a Waters 2695 Millennium system equipped with a photodiode array detector. EI and CI mass spectra were performed on a Trace DSQ GC/MS spectrometer. Data are reported in the form of(m/z). The organocatalysts were prepared according to references [29,30,31,32,33,34,35,36]. All the Michael products were known and confirmed by GC-MS, and usual spectral methods (NMR and IR).

### 3.2. General Procedure for Michael Addition of 1,3-Dicarbonyl Indane Compounds to Nitroolefins

A catalytic amount of Ts-DPEN (10 mol %) was added to a vial containing olefins (0.5 mmol) and 1,3-dicarbonyl compounds (0.55 mmol) in toluene (1 mL). After vigorous stirring at -20 °C or -40 °C for the times shown in the Table, the reaction mixture was poured into an extraction funnel containing brine, diluted with distilled water, and EtOAc. The aqueous phase was extracted with EtOAc. The combined organic phases were dried with Na_2_SO_4_ and the solvent was removed under reduced pressure. The crude product was purified by silica gel column chromatography to furnish the desired Michael products. All the diastereomers could not be separated and were confirmed by GC-MS, NMR and IR, the *ees* of the Michael products were determined by chiral-phase HPLC analysis using a Chiralcel OD-H column and the indicated eluent systems [27,28]. The relative configuration of products was determined by comparison of the ^1^H-NMR spectra and HPLC with literature data [39,40]. 

Methyl 2-(2-nitro-1-phenylethyl)-1-oxo-2,3-dihydro-1H-indene-2-carboxylate (Table 1, entry 1) **6a** [20]: yellow amorphous solid; ^1^H-NMR (CDCl_3_) (ppm): δ = 3.14–3.19 (d, *J* = 17.6 Hz,1H), 3.47–3.51 (d, *J*=17.2 Hz, 1H), 3.70 (s, 3H), 4.47–4.50 (dd, *J* = 11.2, 3.2 Hz,1H), 5.05–5.09 (dd, *J* = 13.6, 3.6 Hz, 1H), 5.17–5.23 (m, 1H), 7.13–7.26 (m, 6H), 7.34–7.38 (t, *J =* 7.6 Hz, 1H), 7.49–7.53 (t, *J* = 7.6 Hz, 1H), 7.76–7.78 (d, *J* = 7.6 Hz, 1H); ^13^C-NMR (CDCl_3_, ppm): δ = 202.1, 199.9, 171.2, 169.9, 152.4, 136.2, 135.9, 135.8, 135.7, 134.8, 134.0, 129.07, 129.02, 128.9, 128.7, 128.4, 128.08, 128.03, 126.1, 125.2, 124.5, 62.8, 61.8, 53.3, 47.6, 47.1, 36.6. GC-MS: m/z :339 (M); HPLC: hexane/2-propanol = 80/20, 0.8 mL/min, λ = 210 nm, retention times: (major enantiomer) 14.8 min, (minor enantiomer) 31.5 min, (minor diastereomers) [13.1, 54.6 min]. 

Methyl 2-(2-nitro-1-p-tolylethyl)-1-oxo-2,3-dihydro-1H-indene-2-carboxylate (Table 1, entry 2) **6b:** ^1^H-NMR (CDCl_3_) (ppm): δ = 2.21 (s, 3H), 3.18–3.27 (d, *J* = 17.2 Hz, 1H), 3.49–3.53 (d, *J* = 17.2 Hz, 1H), 3.71 (s, 3H), 4.46–4.49 (dd, *J* = 11.2, 3.2 Hz, 1H), 5.04–5.08 (dd, *J* = 13.6, 3.2 Hz, 1H), 5.17–5.23 (m, 1H), 7.04–7.43 (m, 7H), 7.78–7.80 (d, *J* = 7.6 Hz, 1H); ^13^C-NMR (CDCl_3_, ppm): δ = 202.1, 199.9, 171.2, 169.9, 152.5, 138.1, 136.2, 135.9, 135.7, 134.8, 134.1, 132.7, 131.7, 129.6, 129.4, 128.9, 128.8, 128.0, 127.9, 126.2, 125.2, 124.5, 62.9, 61.9, 53.2, 47.3, 36.5, 21.0. MS (EI):m/z =353.03; HPLC: hexane/2-propanol = 80/20, 0.8 mL/min, λ = 210 nm, retention times: (major enantiomer) 13.3 min, (minor enantiomer) 28.4 min, (minor diastereomers) [12.5, 48.6 min].

*Methyl 2-(1-(2-chlorophenyl)-2-nitroethyl)-1-oxo-2,3-dihydro-1H-indene-2-carboxylate (Table 1, entry* *3)* **6c:**: ^1^H-NMR (CDCl_3_) (ppm): δ = 3.10–3.17 (dd, *J* = 17.6, 7.6 Hz, 1H), 3.44–3.56 (dd, *J* = 29.6, 17.6 Hz,1H), 3.72 (s, 3H), 5.20–5.44 (m, 3H), 6.89–7.06 (m, 3H) ,7.19–7.51 (m, 4H), 7.75–7.81(dd, *J* = 19.2, 8 Hz, 1H); ^13^C-NMR (CDCl_3_, ppm): δ = 202.6, 171.5, 152.5, 136.3, 135.9, 135.8, 132.9, 130.1, 129.4, 128.4, 128.0, 127.1, 126.0, 124.4, 62.9, 61.2, 53.3, 42.0, 36.7. MS (EI): m.z = 338.01; HPLC: hexane/2-propanol = 80/20, 0.8 mL/min, λ = 210 nm, retention times: (major enantiomer) 11.8 min, (minor enantiomer) 33.6 min, (minor diastereomers) [11.3, 16.5 min].

*Methyl 2-(1-(4-bromophenyl)-2-nitroethyl)-1-oxo-2,3-dihydro-1H-indene-2-carboxylate (Table 1, Entry 4)* **6d:** ^1^H-NMR (CDCl_3_) (ppm): δ = 3.09–3.13 (d, *J* = 17.6 Hz, 1H), 3.48–3.52 (d, *J* = 17.6 Hz, 1H), 3.69 (s, 3H), 4.44–4.47 (dd, *J* = 11.2, 3.6 Hz, 1H), 5.02–5.06 (dd, *J* = 13.2, 3.2 Hz, 1H), 5.17–5.23 (dd, *J* = 13.6, 11.2 Hz, 1H), 7.02–7.04 (d, *J* = 8.4 Hz, 2H), 7.25–7.29 (m, 3H), 7.34–7.42 (m, 1H), 7.53–7.57 (t, *J =* 7.2 Hz, 1H), 7.76–7.78 (d, *J* = 8 Hz,1H); ^13^C-NMR (CDCl_3_, ppm): δ = 201.8, 171.0, 152.2, 136.0, 133.9, 131.9, 130.7, 128.2, 126.2, 124.6, 122.6, 100.0, 61.5, 53.4, 47.0, 36.6; MS (EI): m/z =416.98; HPLC: hexane/2-propanol = 80/20, 1 mL/min, λ = 210 nm, retention times: (major enantiomer) 17.7 min, (minor enantiomer) 41.4 min, (minor diastereomers) [13.2, 78.6 min].

*Methyl 2-(1-(4-methoxyphenyl)-2-nitroethyl)-1-oxo-2,3-dihydro-1H-indene-2-carboxylate (Table 1, Entry 5)* **6e:** ^1^H-NMR (CDCl_3_) (ppm): δ = 3.17–3.26 (t, *J* = 17.6Hz, 1H), 3.48–3.53 (d, *J* = 17.6 Hz, 1H), 3.69–3.75 (dd, *J* = 16, 7.2 Hz, 6H), 4.20–4.47 (m, 1H), 5.03–5.41 (m, 2H), 6.67–6.78 (dd, *J* = 35.2, 8.4 Hz, 2H), 7.07–7.21 (dd, *J* = 46.4, 8.8 Hz, 2H, 7.26–7.60 (m, 3H), 7.69–7.79 (dd, *J* = 34.4, 7.6 Hz, 1H); ^13^C-NMR (CDCl_3_, ppm): δ = 202.2, 200.0, 171.3, 169.9, 159.3, 152.4, 136.2, 135.9, 135.8, 130.2, 130.1, 128.0, 128.1, 127.5, 126.5, 126.1, 125.2, 124.5, 114.2, 114.1, 63.1, 62.0, 55.1, 53.2, 47.0, 36.6; MS (EI): m/z =369.06; HPLC: hexane/2-propanol = 80/20, 0.5 mL/min, λ = 210 nm, retention times: (major enantiomer) 29.6 min, (minor enantiomer) 73.6 min, (minor diastereomers) [28.7, 124.4 min].

*Methyl 2-(1-(4-chlorophenyl)-2-nitroethyl)-1-oxo-2,3-dihydro-1H-indene-2-carboxylate (Table 1, Entry 6)* **6f:** ^1^H-NMR (CDCl_3_) (ppm):δ = 3.09–3.14 (d, *J* = 17.6 Hz, 1H), 3.48–3.52 (d, *J* = 17.6 Hz, 1H), 3.69 (s, 3H), 4.45–4.49 (dd, *J* = 10.8, 3.2 Hz, 1H), 5.03–5.07 (dd, *J* = 13.6, 3.6 Hz, 1H), 5.13–5.19 (dd, *J* = 13.6, 11.2 Hz, 1H), 7.08–7.27 (m, 5H), 7.36–7.56 (m, 2H), 7.76–7.78 (d, *J* = 8 Hz, 1H); ^13^C-NMR (CDCl_3_, ppm): δ = 201.8, 171.0, 152.2, 136.0, 134.4, 133.4, 130.4, 128.9, 128.2, 126.2, 124.5, 61.6, 53.3, 46.9, 36.6; MS (EI): m/z =373.03; HPLC: hexane/2-propanol = 80/20, 0.8 mL/min, λ = 210 nm, retention times: (major enantiomer) 20.6 min, (minor enantiomer) 50.7 min, (minor diastereomers) [14.9, 96.6 min].

*Methyl 2-(1-(2,6-dichlorophenyl)-2-nitroethyl)-1-oxo-2,3-dihydro-1H-indene-2-carboxylate (Table 1, Entry 7)* **6g:** ^1^H-NMR (CDCl_3_) (ppm):δ = 3.05–3.12 (dd, *J* = 17.6, 8.4 Hz, 1H), 3.35–3.60 (m, 2H), 3.72 (s, 3H), 5.14–5.39 (m, 2H), 6.91–7.42 (m, 5H), 7.50–7.55 (dd, *J* = 14.8, 7.6 Hz, 1H), 7.75–7.82 (m, 1H); ^13^C-NMR (CDCl_3_, ppm): δ = 202.3, 199.4, 171.3, 152.3, 136.7, 136.1, 135.5, 134.6, 131.7, 130.0, 128.2, 127.8, 127.5, 126.6, 126.2, 124.7, 124.3, 61.1, 53.4, 41.7, 36.7, 30.3; MS (EI): m/z =407.08; HPLC: hexane/2-propanol = 80/20, 0.8mL/min, λ = 210 nm, retention times: (major enantiomer) 13.0 min, (minor enantiomer) 51.6 min, (minor diastereomers) [10.5 ,21.1 min].

*Methyl 2-(1-(4-fluorophenyl)-2-nitroethyl)-1-oxo-2,3-dihydro-1H-indene-2-carboxylate (Table 1, Entry 8)* **6h:** ^1^H-NMR (CDCl_3_) (ppm):δ = 3.10–3.15 (d, *J* = 17.6 Hz, 1H), 3.47–3.52 (d, *J* = 17.6 Hz, 1H), 3.70 (s, 3H), 4.45–4.49 (dd, *J* = 11.2, 3.6 Hz, 1H), 5.03–5.08 (dd, *J* = 13.6, 3.6 Hz, 1H), 5.14–5.20 (dd, *J* = 13.6, 11.2 Hz, 1H), 6.80–6.86 (m, 2H), 7.10–7.15 (m, 2H), 7.24–7.27 (m, 1H), 7.35–7.39 (m, 1H), 7.51–7.55 (m, 1H), 7.75–7.77 (d, *J* = 8 Hz, 1H). ^13^C-NMR (CDCl_3_, ppm): δ = 201.9,199.8, 171.1, 152.3, 136.0, 130.1, 128.2, 126.1, 124.5,115.6, 61.9, 53.3, 46.9, 36.6; MS (EI): m/z =357.05; HPLC: hexane/2-propanol = 80/20, 0.8mL/min, λ = 210 nm, retention times: (major enantiomer) 17.2 min, (minor enantiomer) 43.7 min, (minor diastereomers) [13.1, 83.3 min].

*Methyl 2-(1-(2-bromophenyl)-2-nitroethyl)-1-oxo-2,3-dihydro-1H-indene-2-carboxylate (Table 1, Entry 9)* **6i:** ^1^H-NMR (CDCl_3_) (ppm):δ = 3.13–3.20 (dd, *J* = 17.6, 8.8 Hz, 1H), 3.40–3.49 (m, 1H), 3.73 (s, 3H), 5.20–5.42 (m, 3H), 6.93–7.05 (m, 2H) , 7.19–7.68 (m, 5H), 7.75–7.81 (m, 1H); ^13^C-NMR (CDCl_3_, ppm): δ = 202.6, 199.4, 171.5, 152.5, 136.3, 135.8, 135.5,134.6, 133.5, 129.6, 128.4, 128.0, 127.8, 126.6,126.0, 124.8,124.3, 62.9, 61.2, 53.3, 44.8, 36.7, 30.3; MS (EI): m/z = 418.21; HPLC: hexane/2-propanol = 80/20, 0.8 mL/min, λ = 210 nm, retention times: (major enantiomer) 12.3 min, (minor enantiomer) 35.8 min, (minor diastereomers) [11.8, 16.0 min].

*Methyl 2-(1-(4-cyanophenyl)-2-nitroethyl)-1-oxo-2,3-dihydro-1H-indene-2-carboxylate (Table 1, Entry 10)* **6k:** ^1^H-NMR (CDCl_3_) (ppm): δ = 3.05–3.09 (d, *J* = 17.6 Hz, 1H), 3.50–3.55 (d, *J* = 17.6 Hz, 1H), 3.70 (s, 3H), 4.53–4.56 (dd, *J* = 11.2, 3.6 Hz, 1H), 5.07–5.12 (dd, *J* = 14, 3.6 Hz, 1H), 5.16–5.22 (dd, *J* = 14, 11.2 Hz,1H), 7.25–7.46 (m, 6H), 7.53–7.58 (m, 1H), 7.76–7.78(d, *J*= 8Hz, 1H); ^13^C-NMR (CDCl_3_, ppm): δ = 201.3, 170.7, 151.9, 140.5, 136.2, 135.9, 132.4, 129.9, 128.4, 126.2, 124.7, 118.0, 112.5, 61.4, 53.4, 47.4, 36.6; MS (EI): m/z =363.95; HPLC: hexane/2-propanol = 80/20, 0.8 mL/min, λ = 210 nm, retention times: (major enantiomer) 47.2 min, (minor enantiomer) 101.4 min, (minor diastereomers) [24.8, 182.7 min].

*Methyl 2-(1-(2-bromophenyl)-2-nitroethyl)-4-methyl-1-oxo-2,3-dihydro-1H-indene-2-carboxylate (Table 1, Entry 11)* **6l:** ^1^H-NMR (CDCl_3_) (ppm):δ = 2.37 (s, 3H), 3.06-3.13 (dd, *J* = 17.6, 10.8 Hz, 1H), 3.37–3.47 (dd, *J* = 24, 17.6 Hz, 1H), 3.72 (s, 3H), 5.19–5.41 (m, 3H),6.95–7.17 (m, 3H) , 7.30–7.34 (t, *J* = 8 Hz ,1H), 7.47–7.68 (m, 3H); ^13^C-NMR (CDCl_3_, ppm): δ = 202.6, 200.2, 171.6, 150.0, 138.3, 138.1, 137.5, 137.1, 136.5, 134.7, 133.6, 133.5, 129.7, 129.6, 128.4, 127.8, 127.4, 126.0, 125.7, 125.3, 124.6,124.2 ,63.2, 61.5, 53.3, 44.7, 36.4, 29.7, 21.1; MS (EI): m/z = 352.23; HPLC: hexane/2-propanol = 60/40, 0.4mL/min, λ = 210 nm, retention times: (major enantiomer) 15.5 min, (minor enantiomer) 43.8 min, (minor diastereomers) [16.1, 17.7 min].

*Methyl 2-(1-(2-bromophenyl)-2-nitroethyl)-4-methoxy-1-oxo-2,3-dihydro-1H-indene-2-carboxylate (Table 1, Entry 12)* **6m:** ^1^H-NMR (CDCl_3_) (ppm):δ = 3.04–3.08 (d, *J* = 17.6 Hz, 1H), 3.22–3.36 (m, 1H), 3.49–3.54 (dd, *J* = 17.2, 4 Hz, 1H), 3.79 (s, 3H), 3.90 (s, 3H),5.17–5.43 (m, 2H), 6.92–7.27 (m, 5H) , 7.67–7.75 (m, 2H); ^13^C-NMR (CDCl_3_, ppm): δ = 200.3, 197.4, 171.7,169.9, 156.7, 155.7, 134.8, 133.4, 129.6, 128.5, 127.8, 126.4, 126.1, 116.3, 116.0, 109.6, 108.9, 63.1, 61.4, 55.8, 53.4, 44.7, 36.6, 30.3, 25.4; MS (EI): m/z = 368.02; HPLC: hexane/2-propanol = 80/20, 0.8 mL/min, λ = 210 nm, retention times: (major enantiomer) 19.5 min, (minor enantiomer) 32.6 min, (minor diastereomers) [14.9, 17.2 min].

*Methyl 5-bromo-2-(1-(2-bromophenyl)-2-nitroethyl)-1-oxo-2,3-dihydro-1H-indene-2-carboxylate (Table 1, Entry 13)* **6n:** ^1^H-NMR (CDCl_3_) (ppm):δ = 3.11–3.18 (dd, *J* = 17.6, 8.8 Hz, 1H), 3.41–3.54 (m, 1H), 3.73 (s, 3H), 5.18–5.40 (m, 3H), 7.05–7.09 (m, 2H), 7.47–7.74 (m, 5H); ^13^C-NMR (CDCl_3_, ppm): δ = 201.4, 198.9, 171.1, 153.9, 135.1, 134.4, 133.7, 132.0, 131.8, 131.4, 129.8, 129.4, 128.3, 127.9, 127.4, 126.6, 125.4, 62.9, 61.3, 53.4, 44.5, 36.2, 31.6, 25.4; MS (EI): m/z = 418; HPLC: hexane/2-propanol = 80/20, 0.8 mL/min, λ = 210 nm, retention times: (major enantiomer) 15.0 min, (minor enantiomer) 37.0 min, (minor diastereomers) [12.7, 18.4 min].

*Methyl 2-(1-(2-chlorophenyl)-2-nitroethyl)-5-methyl-1-oxo-2,3-dihydro-1H-indene-2-carboxylate (Table 1, Entry 14)* **6o:** ^1^H-NMR (CDCl_3_) (ppm):δ = 2.37 (s, 3H), 3.04–3.10 (dd, *J* = 17.6, 9.2 Hz, 1H), 3.37–3.49 (dd, *J* = 30, 17.6 Hz, 1H), 3.72 (s, 3H), 5.20–5.41 (m, 3H), 6.93–7.10 (m, 3H),7.30–7.67 (m, 4H); ^13^C-NMR (CDCl_3_, ppm): δ = 202.6, 200.2, 171.6, 149.9, 138.4, 138.1, 137.5, 137.1, 136.5, 136.0, 130.0, 130.2, 129.4, 128.4, 127.8, 127.1, 126.0, 125.7, 125.3, 124.2, 63.2, 61.5, 53.3, 42.0, 36.4, 21.1; MS (EI): m/z = 351.94; HPLC: hexane/2-propanol = 60/40, 0.4mL/min, λ = 210 nm, retention times: (major enantiomer) 15.0 min, (minor enantiomer) 42.5 min, (minor diastereomers) [15.8, 18.2 min].

*Methyl 2-(1-(2-chlorophenyl)-2-nitroethyl)-5-methoxy-1-oxo-2,3-dihydro-1H-indene-2-carboxylate (Table 1, Entry 15)* **6p:** ^1^H-NMR (CDCl_3_) (ppm):δ = 3.01–3.06 (d, *J* = 17.6 Hz, 1H), 3.28–3.40 (m, 1H), 3.49–3.54 (dd, *J* = 17.2, 4 Hz, 1H), 3.79 (s, 3H), 3.90 (s, 3H), 5.18–5.43 (m, 2H), 6.85–7.40 (m, 5H), 7.67–7.74 (m, 2H); ^13^C-NMR (CDCl_3_, ppm): δ = 200.2, 197.5, 171.8, 169.9, 156.7, 155.7, 135.9, 133.0, 130.0, 129.6, 129.3, 128.5, 127.1, 127.0, 126.4, 126.0, 116.3, 116.0, 109.6, 109.0, 61.4, 55.8, 53.4, 41.9, 36.6, 30.3, 25.4; MS (EI): m/z = 367.96; HPLC: hexane/2-propanol = 80/20, 0.8 mL/min, λ =210 nm, retention times: (major enantiomer)15.0 min, (minor enantiomer) 36.3 min, (minor diastereomers) [12.5, 20.1 min].

*Methyl 5-bromo-2-(1-(2-chlorophenyl)-2-nitroethyl)-1-oxo-2,3-dihydro-1H-indene-2-carboxylate (Table 1, Entry 16)* **6q:** ^1^H-NMR (CDCl_3_) (ppm):δ = 3.08–3.15 (dd, *J* = 18, 8Hz, 1H), 3.41–3.52 (dd, *J* = 28, 18Hz, 1H), 3.73 (s, 3H), 4.58–4.62 (m, 1H), 5.18–5.40 (m, 2H), 6.96–7.11 (m, 2H), 7.33–7.67 (m, 5H); ^13^C-NMR (CDCl_3_, ppm): δ = 201.4, 198.9, 171.1, 153.9, 135.9, 135.1, 132.7, 131.8, 131.4, 130.3, 129.6, 129.4, 128.3, 127.3, 126.6, 125.5, 62.9, 61.3, 53.4, 41.8, 36.2, 29.8, 25.4; MS (EI): m/z = 416.4; HPLC: hexane/2-propanol = 80/20, 0.8 mL/min, λ = 210 nm, retention times: (major enantiomer)15.0 min, (minor enantiomer) 36.3 min, (minor diastereomers) [12.5, 20.1min].

## 4. Conclusions

In summary, a study of a dozen of different organocatalysts in the Michael reaction of 1,3-dicarbonyl indane compounds to nitrostyrenes has identified a satisfactory primary amine organocatalyst. A primary amine-based catalyst, Ts-DPEN, bearing an amino sulfonamide moiety and with a primary amino group on a chiral scaffold was found to be a simple and efficient bifunctional organocatalyst for the asymmetric Michael addition of 1,3-dicarbonyl indane compounds to nitrostyrenes, which gave highly functional Michael adducts with quaternary stereocenters in good enantioselectivities (up to 92:8 er) and good *dr* (up to 81:15 dr). Surprisingly, the introduction of thiourea, amide, phenol, tertiary amine, and imine groups in Ts-DPEN did not lead to higher enantioselectvities, indicating the importance of the primary amine group in controlling the enantioselectivity in this Michael reaction. A reasonable catalytic reaction mechanism was proposed on the basis of the experimental results.
